# Supplementary material for: Comparing and scaling fMRI features for brain-behavior prediction
Source: Imaging Neurosci (Camb). 2025 Sep 12;3:IMAG.a.141. doi: 10.1162/IMAG.a.141 (PMC12434381; doi:10.1162/IMAG.a.141)
Supplement: Supplementary Material [file IMAG.a.141_supp.pdf]

Supplementary Information

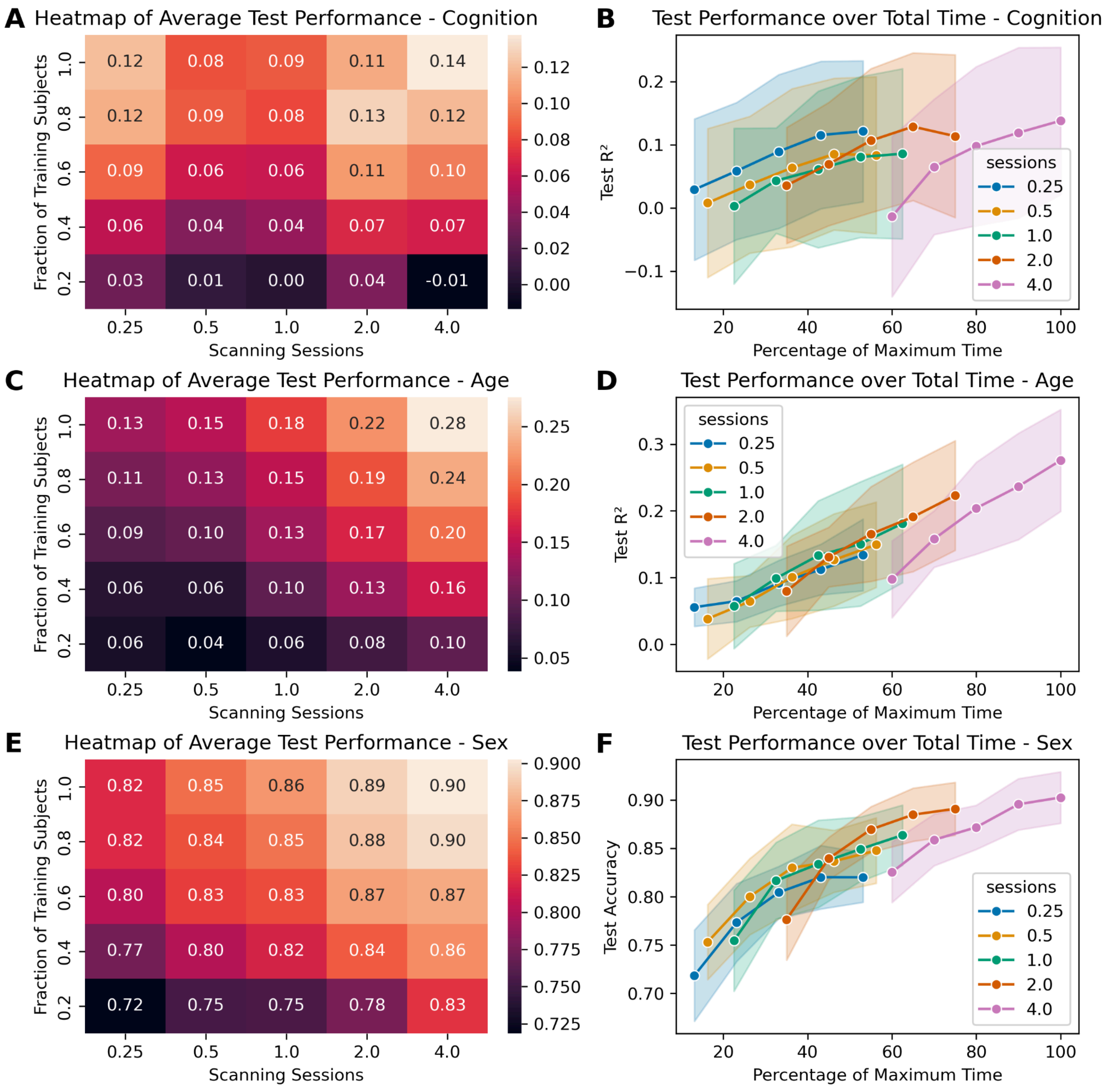

Figure S1: Scaling effects predicting different targets from FC using elastic-net regression (for cognition and age) or elastic-net classifier (for sex) as the model. (A) Heatmap showing the average coefficient of determination (R^2) for each combination of fraction of training subjects (rows) and number of scanning sessions (columns) for cognition. (B) Test performance (R^2) scaling curve over the maximum available scan time for predicting cognition. 100 % represents four scanning sessions with all available training subjects. Shown is the mean performance for each number of sessions, with the SD as the error band. (C) Heatmap for age. (D) Scaling curve for age. (E) Heatmap for sex (accuracy). (F) Scaling curve for sex (accuracy).
